# Factors Impacting Stent Thrombosis in Patients With Percutaneous Coronary Intervention and Coronary Stenting: A Systematic Review and Meta-Analysis

**DOI:** 10.7759/cureus.23973

**Published:** 2022-04-09

**Authors:** Nso Nso, Mahmoud Nassar, Milana Zirkiyeva, Yolanda Mbome, Anthony Lyonga Ngonge, Solomon O Badejoko, Shahzad Akbar, Atika Azhar, Sofia Lakhdar, Laura M Guzman Perez, Yousef Abdalazeem, Vincent Rizzo, Most Munira

**Affiliations:** 1 Internal Medicine, Icahn School of Medicine at Mount Sinai/NYC Health+Hospitals Queens, New York City, USA; 2 Internal Medicine, Richmond University Medical Center, New York City, USA; 3 Medicine, Howard University Hospital, Washington, D.C., USA; 4 Internal Medicine, St. Joseph’s Medical Center, Stockton, USA; 5 Internal Medicine, Kettering Health, Dayton, USA; 6 Internal Medicine, Upstate University Hospital, Syracuse, USA; 7 Medicine, Icahn School of Medicine at Mount Sinai/NYC Health+Hospitals Queens, New York City, USA; 8 Internal Medicine, Icahn School of Medicine at Mount Sinai, Queens Hospital Center, New York City, USA; 9 Emergency Medicine, East and North Hertfordshire NHS Trust/North Hertfordshire College (NHC), Stevenage, GBR; 10 Cardiology/Medicine, Weil Cornell Medicine, New York City, USA; 11 Cardiology, Icahn School of Medicine at Mount Sinai/NYC Health+Hospitals Queens, New York City, USA

**Keywords:** non-st segment elevation myocardial infarction (nstemi), st-elevation myocardial infarction (stemi), bare-metal stents, drug-eluting stents, acute coronary syndrome, coronary stenting, percutaneous coronary intervention, stent thrombosis

## Abstract

Stent thrombosis (ST) is a frequently reported complication in cardiac patients with percutaneous coronary intervention (PCI) that adversely impacts their prognostic outcomes. Medical literature reveals several baseline characteristics of PCI patients that may predict their predisposition to ST and its potential complications. Our systematic review and meta-analysis aimed to determine the diagnostic significance of these baseline parameters in terms of determining the risk of ST among adult patients with PCI.

We statistically evaluated 18 baseline characteristics of more than 15,500 PCI patients to delineate their stent thrombosis attribution. We included a number of articles focusing on baseline parameters in-stent thrombosis-related PCI scenarios. We explored the articles of interest based on inclusion/exclusion parameters across PubMed, JSTOR, Cochrane library, Google Scholar, and Embase. Medical subject headings (MeSH) words included “stent thrombosis,” “percutaneous coronary intervention,” and “coronary stenting.” We extracted the research articles published between 2005 and 2021 on April 20, 2021. The included studies also focused on procedures and clinical factors concerning their association with PCI-related ST.

Our findings ruled out the progression of abnormal left ventricular ejection fraction (LVEF)-related stent thrombosis in PCI patients (odds ratio {OR}: 9.68, 95% CI: 1.88-49.90, p=0.007). We found an insignificant clinical correlation between stent thrombosis and PCI in the setting of acute coronary syndrome (ACS). Our study outcomes further revealed the absence of stent thrombosis in PCI patients with antiplatelet prescription (OR: 32.42, 95% CI: 21.28-49.39). The findings affirmed the absence of ST in PCI patients receiving aspirin therapy (OR: 32.77, 95% CI: 18.73-57.34; OR: 4.59, 95% CI: 1.97-10.73). The majority of the included studies negated the clinical correlation of stent thrombosis with diabetes mellitus in the setting of PCI (OR: 0.49, 95% CI: 0.06-3.78). Our study did not reveal statistically significant results based on stent thrombosis in PCI patients with drug-eluting stents (OR: 2.91, 95% CI: 0.35-24.49). The findings also did not reveal the impact of cardiac biomarker elevation on stent thrombosis in PCI patients (OR: 8.42, 95% CI: 2.54-27.98, p=0.0005). Eight studies revealed a statistically insignificant correlation between myocardial infarction and stent thrombosis in PCI scenarios (OR: 2.69, 95% CI: 0.89-8.11, p=0.08). The clinical correlation between PCI and stent thrombosis/major bleeding in the setting of hypertension also proved statistically insignificant at 0.67 (OR: 1.31, 95% CI: 0.38-4.51, p=0.97). The study findings did not correlate mean body mass index and multivessel coronary artery disease with ST in PCI scenarios (OR: 1.98, 95% CI: 0.02-239.58, p=0.78; OR: 1.09, 95% CI: 0.58-2.04, p=0.80). Only two studies revealed statistically significant results confirming stent thrombosis in PCI patients with a prior history of PCI (OR: 0.49, 95% CI: 0.23-1.06; OR: 0.33, 95% CI: 0.02-5.59; p=0.03).

Our findings question the clinical significance of baseline characteristics in terms of predicting stent thrombosis in PCI patients. The results support the requirement of future studies to investigate complex interactions between procedural, medicinal, genetic, and patient-related factors contributing to the development of stent thrombosis in PCI patients.

## Introduction and background

Percutaneous coronary intervention (PCI), coronary stenting, or percutaneous transluminal coronary angioplasty (PTCA) help dilate the stenosed or blocked coronary arteries to enhance the myocardial blood supply in the least restrictive manner [1]. The diagnostic indications of PCI include angina symptomatology, shortness of breath, fatigue, atypical chest pain, abnormal stress test results, and preoperative state [2]. Coronary stenting minimizes the angina symptoms and improves the prognostic outcomes in patients with a clinical history of ischemia [3]. PCI utilizes bare-metal/drug-eluting stents, bioresorbable scaffold systems, or drug-eluting balloons. PCI risk factors include hemodynamic status, coronary anatomy (and its complexities), comorbidities, and patient attributes [4]. The potential complications of PCI include aortic/coronary artery rupture/dissection, bleeding, bacteremia, renal failure, stroke, myocardial infarction (MI), and stent thrombosis (ST) [5]. 

ST is an abnormal outcome of PCI that abruptly closes the coronary vessels due to the thrombotic obstruction of the coronary stent [6]. It often develops due to premature clopidogrel discontinuation, stent under-sizing, coronary dissection, postprocedural thrombolysis in myocardial infarction (TIMI) flow, comorbid lesions, malignancy, discontinuation of aspirin, and impaired left ventricular ejection fraction (LVEF) [7]. The reduction in the concentration of tissue plasminogen activator, prostacyclin I2, and nitric oxide also triggers prothrombotic states in PCI patients [8]. 

The clinical studies report a 0.5-2.2% incidence of ST in patients with PCI. The fatal outcomes of ST in PCI patients include MI and cardiovascular death [9]. The multifactorial causes of ST include patient-related attributes, lesion/procedure complexity, and thrombogenicity. The PCI patients who receive dual antiplatelet therapy experience a marked reduction in their ST incidence attributed to 0.2-0.6% [6]. The ST events in PCI patients with drug-eluting stents trigger at three months compared to 30-days in patients with bare-metal stents. The previous meta-analysis by D'Ascenzo et al., in 2013, defined stent length/number, the extent of coronary disease, and antiplatelet therapy discontinuation as the prominent risk factors for ST [10]. The network meta-analysis by Philip et al., in 2016, affirmed a marked reduction in ST events in PCI patients who received drug-eluting stents [11]. The systematic review and meta-analysis by Yuan and Xu, in 2018, alternatively confirmed a similar ST rate among diabetic and non-diabetic PCI patients [12]. The study by Hara et al., in 2021, precisely underscored the lack of adequate prediction models to discriminate between ST risk scores and bleeding predisposition in patients undergoing PCI [13]. To the best of our knowledge, no study examined the incidence of ST events in PCI patients based on their baseline characteristics until April 20, 2021. 

This systematic review and meta-analysis is the first study of its type that investigates the possible role of baseline and procedural and lesion characteristics of PCI patients in predicting the incidence of their ST events. We accordingly quantified ST/no-ST events in PCI patients after pooling their baseline/procedural/lesion characteristics. 

Methodology

We followed the Preferred Reporting Items for Systematic Reviews and Meta-Analyses (PRISMA) framework for our systematic review and meta-analysis [14]. The primary analysis investigated incidence/no incidence of ST events for each of the following baseline characteristics of 15,500 patients: abnormal left ventricular ejection fraction, acute coronary syndrome (ACS) status, antiplatelet prescription, aspirin prescription, diabetes mellitus, drug-eluting stent status, dyslipidemia, elevated cardiac biomarkers, heart failure, history of MI or coronary artery disease (CAD), hypertension, major bleeding, mean body mass index (BMI), percutaneous coronary intervention (PCI) for multivessel coronary artery disease (CAD), prior history of percutaneous coronary intervention (PCI), renal insufficiency, smoking history, and ST-segment elevation myocardial infarction (STEMI).

The subgroup analysis examined the following procedural and lesion characteristics of PCI patients in the context of their ST/no-ST events, anterior Q waves, atrial fibrillation, bivalirudin monotherapy, dual antiplatelet therapy (DAPT), in-stent diameter, lesion length/diameter, lesion location (ostial or bifurcation lesion), lesion target vessel (left anterior descending coronary artery), lesion target vessel (left circumflex coronary artery), lesion target vessel (right coronary artery), number of stents, stent diameter (pre-procedure diameter stenosis), stent length, thrombolysis in myocardial infarction (TIMI) flow (coronary artery flow in acute coronary syndromes).

The medical subject headings (MeSH) words included “stent thrombosis,” “percutaneous coronary intervention,” and “coronary stenting.” We extracted the research articles (published between 2005 and 2021) on April 20, 2021. We examined the risk of publication bias in findings by constructing funnel plots for the outcome variables. 

Inclusion and exclusion criteria 

We explored a range of articles based on randomized (single-center/multicenter) open-label trials, case-control/observational studies, and prospective/retrospective (cohort) studies across PubMed, JSTOR, Cochrane library, Google Scholar, and Embase. We considered studies (published between 2005 and 2021) that focused on the predictors or risk factors for ST in PCI scenarios. We excluded studies that analyzed ST episodes in patients with active pregnancy and active tumors. We also did not consider studies that examined ST in PCI scenarios for patients below 18 years of age. We further excluded studies with reduced power, statistically insignificant findings, and validity/reliability issues. 

Study selection parameter

**Figure 1:**
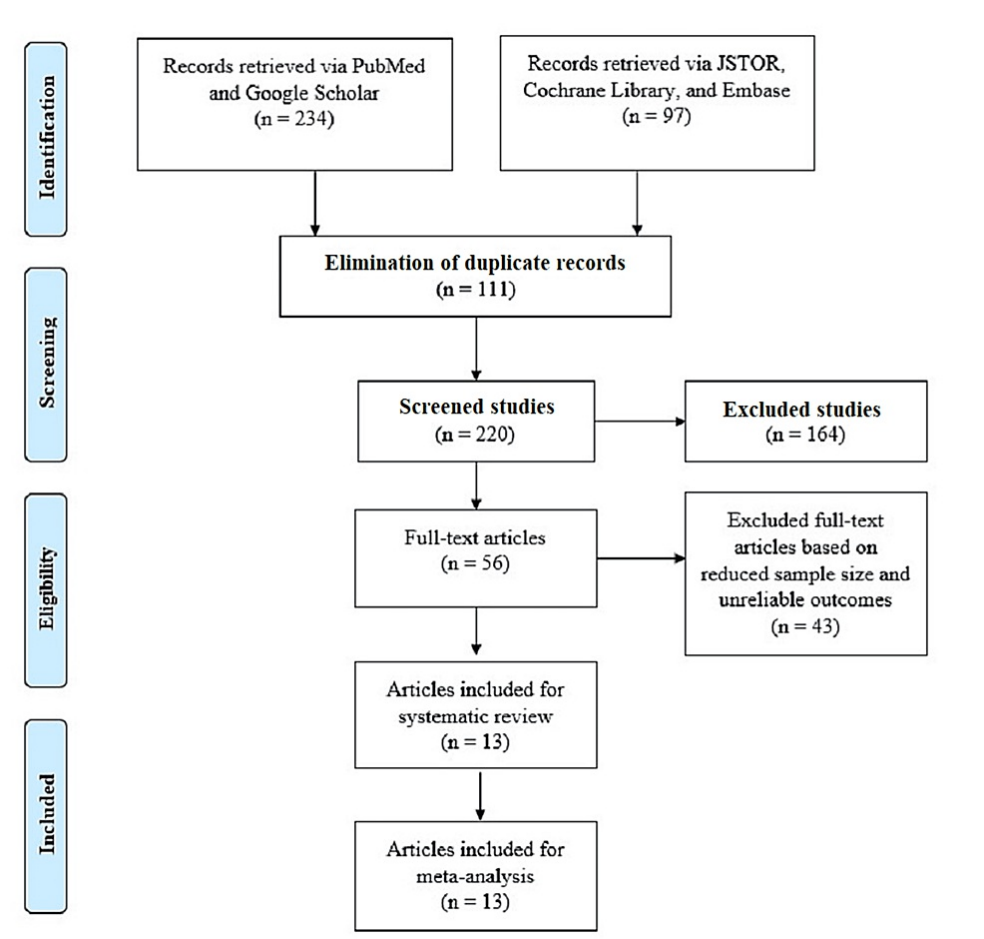
PRISMA flow chart PRISMA: Preferred Reporting Items for Systematic Reviews and Meta-Analyses

We utilized a PRISMA flow chart to explore full-text studies documenting baseline characteristics of PCI patients with ST (Figure 1) [15]. We initially explored 331 studies based on the inclusion and exclusion parameters. The screening of 220 studies followed the elimination of 111 duplicate records. We further excluded 207 studies due to their unreliable outcomes, limited sample sizes, and unavailability of full-text articles. We finally included 13 full-text articles matching the selection criteria for our systematic review and meta-analysis. 

Data collection 

We utilized MS Excel-365 to record the baseline characteristics and ST events of patients with PCI. Two of the authors recorded data on the worksheet from the full-text studies. Double-data entry checks and auditing of the workbook excluded the risk of data collection errors and processing inadequacies. One author examined the missing summary statistics and transformed the forest/funnel plots into a presentable format. Two authors validated data transfers and conversions from the excel sheet to Cochrane’s Review Manager 5.4 [16].

Data analysis 

We utilized the RevMan version 5.4 to categorically formulate forest plots of 18 baseline characteristics from the included studies [16]. The forest plots determined ST and no-ST events for each of the study end-points. The effect sizes, p-values, tau-square (τ^2^), chi-square (χ^2^), and I^2^ values determined the statistical significance and heterogeneity of the study results. The no-effect mean vertical line helped evaluate the strength of evidence from the included studies. The forest plots comparatively assessed the incidence of ST and no-ST in PCI scenarios. The funnel plots assisted in tracking systematic heterogeneity and publication bias in the study outcomes. The heterogeneity classification was based on I^2^ ranges of 0-50% (for low heterogeneity), 51-80% (for moderate heterogeneity), and 81-100% (for high heterogeneity). The odds ratios within their 95% confidence interval (CI) determined the statistical significance of the study results.

## Review

Results

Table 1 comprehensively summarizes the overall findings from the included studies. Table 2 shows the baseline characteristics of the PCI patients with and without ST. 

**Table 1:** Summary of the findings PCI: percutaneous coronary intervention; ACF: acute heart failure; STEMI: ST-segment elevation myocardial infarction; ST: stent thrombosis; AHF: acute heart failure; LVEF: left ventricular ejection fraction; ACS: acute coronary syndrome; CAD: coronary artery disease; MI: myocardial infarction; GPI: glycoprotein IIb/IIIa inhibitor; CABG: coronary artery bypass grafting

Study	Mean age (years)	Male/female (n, %)	Hypertension (n, %)	Hypercholesterolemia/hyperlipidemia/dyslipidemia (n, %)	Current smokers (n, %)	Mean BMI	Mean Cr-Cl/renal insufficiency (mL/min)	Mean serum creatinine (mg/dL)	Diabetes mellitus (n, %)	Prior history of CAD/MI/CABG (n, %)	Prior history of PCI (n, %)	DAPT at discharge (n, %)	Aspirin	Clopidogrel/ticagrelor	Major bleeding event (n, %)	Bare metal stents	Drug-eluting stents	Events
Saleh et al. (2016) [9]	52.9	37 (79)	26 (62)	21 (45)	23 (49)	30.2	129	0.84	30 (64)	16 (34)	14 (29.8)	NA	46 (97.9)	45 (95.7)	0	NA	NA	Stent thrombosis (n=47)
	58.4	1893 (80)	1483 (62)	1164 (49)	1035 (44)	28.0	100	1.01	1273 (54)	848 (35.6)	527 (24)	NA	2356 (99)	2347 (98.7)	23 (0.97)	NA	NA	No stent thrombosis (n=2379)
Dangas et al. (2011) [17]	57.9 (50.8 -65.9	75.9 (104/137)	56.2 (77/137)	48.2 (66/137)	60.3 (82/136)	27.7 (24.7-30.7)	5.1 (7/137)	NA	19.7 (27/137)	1.5 (2/137)	20.4 (28/137)	NA	29.9 (41/137)	55.5 (76/137	NA	26.3 (35/133)	72.2 (96/133)	Stent thrombosis (n=137)
	59.9 (52.4 -69.7)	77.3 (2369/3065)	51.8 (1588/3063)	42.3 (1296/3063)	46.9 (1433/3053)	27.1 (24.5-30.1)	2.7 (82/3062)	NA	16.1 (493/3063)	2.5 (77/3063	9.5 (290/3062)	NA	25.6 (784/3061)	63.8 (1955/3063)	NA	27.5 (826/3000)	72.2 (2165/3000)	No stent thrombosis (n=3065)
Silva et al. (2018) [18]	64.8±11.8	46 (70.8)	59 (92.2)	23 (27.7)	28 (57.1)	27.8±4.77	NA	NA	24 (38.7)	NA	NA	NA	14 (29.7)	7 (10.8)	NA	55 (90.2)	2 (3.3)	Stent thrombosis (n=65)
	64.8±12.7	138 (70.8)	146 (76.4	30 (17.5)	95 (64.6)	26.7±3.77	NA	NA	54 (29.5)	NA	NA	NA	11 (8.8)	7 (3.9)	NA	170 (93.4)	5 (2.7)	No stent thrombosis (n=195)
Auffret et al. (2016) [19]	68.0 (58.0-78.0)	234 (31.8)	360 (48.8	325 (44.1)	222 (30.1)	NA	NA	NA	88 (11.9)	9 (1.2)	NA	586 (88.5)	636 (96.1)	414 (62.5)	NA	NA	133 (20.3)	Stent thrombosis (n=737)
	68.0 (58.0-78.0)	252 (34.2)	355 (48.2)	329 (44.6)	220 (29.9	NA	NA	NA	90 (12.2)	7 (0.9	NA	667 (92.1)	697 (96.3)	427 (59.0)	NA	NA	159 (24.1)	No stent thrombosis (n=737)
Zwart et al. (2017) [20]	61.0±11.8	108/437 (24.7)	NA	NA	NA	NA	NA	NA	NA	500/866 (57.7)	NA	NA	NA	NA	NA	270 (61.8)	152 (34.8)	Stent thrombosis (n=437)
	62.3±11.7	235/862 (27.3)	NA	NA	NA	NA	NA	NA	NA	252/437 (57.7	NA	NA	NA	NA	NA	614 (70.9)	238 (27.5)	No stent thrombosis (n=866)
Rozemeijer et al. (2019) [21]	64.9±11.5	43 (78.2)	29 (52.7)	27 (49.1)	23 (41.8)	27.3±4.4	NA	10 (18.2)	16 (29.1)	6 (10.9)	29 (52.7)	NA	NA	NA	NA	26 (47.2)	29 (52.7)	Stent thrombosis (n=55)
	65.6±14.7	167 (75.9)	111 (51.8)	91 (41.3)	77 (35.0)	26.7±4.5	NA	44 (20.0)	40 (18.2)	21 (9.5)	24 (43.6)	NA	NA	NA	NA	88 (40.0)	132 (60.0)	No stent thrombosis (n=220)
Le Feuvre et al. (2008) [22]	60±14	49 (92)	28 (54)	NA	27 (52)	NA	6 (11)	NA	20 (38)	34 (65)/4 (8)	28 (54)	NA	NA	NA	NA	36/52	16 (31)	Stent thrombosis (n=52)
	63±11	2832 (80)	1799 (51)	NA	1353 (38)	NA	560 (16)	NA	1105 (31)	772 (22)/276 (8)	928 (26)	NA	NA	NA	NA	NA	1245 (35)	No stent thrombosis (n=3527)
Lemesle et al. (2008) [23]	65.5±2.3	26 (66.7)	23 (59)	24 (61.5)	20 (51.3)	25.4 6±0.6	7 (17.9)	NA	11 (28.2)	NA	NA	NA	NA	NA	NA	NA	9 (23.1)	Stent thrombosis (n=39)
	67.1 6±2.8	18 (72)	15 (60)	16 (64)	13 (52)	24.5 6±0.8	4 (16)	NA	6 (24)	NA	NA	NA	NA	NA	NA	NA	6 (24)	No stent thrombosis (n=25)
Mishkel et al. (2008) [24]	56.6±16.3	27 (79.4)	NA	NA	19 (55.9)	13 (38.2)	NA	NA	10 (29.4)	9 (26.5)	17 (50.0)	NA	NA	NA	NA	NA	NA	Stent thrombosis (n=34)
	66.7±12.2	3302 (63.2)	NA	NA	1235 (23.6)	2671 (51.1)	NA	NA	1509 (28.9)	1220 (23.3)	1481 (28.3)	NA	NA	NA	NA	NA	NA	No stent thrombosis (n=5225)
Balghith et al. (2013) [25]	59	19 (100)	8 (42)	19 (52)	7 (37)	NA	NA	NA	9 (47)	NA	NA	NA	NA	NA	NA	4 (21)	15 (79)	Stent thrombosis (n=19)
Clemmensen et al. (2015) [26]	63 (54, 72)	5 (35.7)	2 (14.3)	3 (21.4)	3 (21.4)	NA	NA	NA	1 (7.1)	0 (0.0)	0 (0.0	NA	14 (100.0)	5/13 (38.5)	NA	NA	NA	Stent thrombosis (n=14)
	61 (62, 71	518 (23.7	961 (44.0)	812 (37.2)	922 (42.3)	NA	NA	NA	295 (13.5)	193 (8.8)	205 (9.4)	NA	2181/2183 (99.9	1064/2091 (50.9)	NA	NA	NA	No stent thrombosis (n=2184)
Driesman et al. (2015) [27]	57.4	8 (88.9)	3 (33.3)	3 (33.3)	3 (33.3)	NA	NA	NA	2 (22.2)	2 (22.2)	NA	NA	1 (11.1)	1 (11.1)	NA	5 (55.6)	3 (33.3)	Stent thrombosis (n=9)
	62.4	186 (70.7)	153 (58.2)	136 (51.9)	141 (56.6)	NA	NA	NA	44 (16.7)	57 (21.7)	NA	NA	88 (34.0)	16 (6.2)	NA	102 (38.8)	148 (56.3)	No stent thrombosis (n=263)
Brener et al. (2013) [28]	61.4 (52.4-69.5)	74.5 (35/47)	57.4 (27/47)	47.8% (22/46)	NA	NA	NA	86 (64-106)	17.0 (8/47)	14.9 (7/47)	17.0 (8/47)	NA	NA	NA	NA	34 (16/47)	66.0 (31/47)	Stent thrombosis (n=47)
	60.6 (52.7-70.0)	73.5 (4812/6544)	61.1% (3990/6529)	52.5% (3416/6501)	NA	NA	NA	90 (69-116)	24.2 (1581/6528)	22.7 (1463/6452)	29.3 (1913/6525)	NA	NA	NA	NA	19.1 (1248/6544)	80.9 (5296/6544)	No stent thrombosis (n=6544)

**Table 2:** Baseline characteristics of PCI patients with and without stent thrombosis BMI: body mass index; MI: myocardial infarction; PCI: percutaneous coronary intervention; CAD: coronary artery disease; DAPT: dual antiplatelet therapy; Cr-Cl: creatinine clearance (mL/minute); CABG: coronary artery bypass graft; NA: not available

Study	Mean age (years)	Male/female (n, %)	Hypertension (n, %)	Hypercholesterolemia/hyperlipidemia/dyslipidemia (n, %)	Current smokers (n, %)	Mean BMI	Mean Cr-Cl/renal insufficiency (mL/min)	Mean serum creatinine (mg/dL)	Diabetes mellitus (n, %)	Prior history of CAD/MI/CABG (n, %)	Prior history of PCI (n, %)	DAPT at discharge (n, %)	Aspirin	Clopidogrel/ticagrelor	Major bleeding event (n, %)	Bare metal stents	Drug-eluting stents	Events
Saleh et al. (2016) [9]	52.9	37 (79)	26 (62)	21 (45)	23 (49)	30.2	129	0.84	30 (64)	16 (34)	14 (29.8)	NA	46 (97.9)	45 (95.7)	0	NA	NA	Stent thrombosis (n=47)
	58.4	1893 (80)	1483 (62)	1164 (49)	1035 (44)	28.0	100	1.01	1273 (54)	848 (35.6)	527 (24)	NA	2356 (99)	2347 (98.7)	23 (0.97)	NA	NA	No stent thrombosis (n=2379)
Dangas et al. (2011) [17]	57.9 (50.8 -65.9	75.9 (104/137)	56.2 (77/137)	48.2 (66/137)	60.3 (82/136)	27.7 (24.7-30.7)	5.1 (7/137)	NA	19.7 (27/137)	1.5 (2/137)	20.4 (28/137)	NA	29.9 (41/137)	55.5 (76/137	NA	26.3 (35/133)	72.2 (96/133)	Stent thrombosis (n=137)
	59.9 (52.4 -69.7)	77.3 (2369/3065)	51.8 (1588/3063)	42.3 (1296/3063)	46.9 (1433/3053)	27.1 (24.5-30.1)	2.7 (82/3062)	NA	16.1 (493/3063)	2.5 (77/3063	9.5 (290/3062)	NA	25.6 (784/3061)	63.8 (1955/3063)	NA	27.5 (826/3000)	72.2 (2165/3000)	No stent thrombosis (n=3065)
Silva et al. (2018) [18]	64.8±11.8	46 (70.8)	59 (92.2)	23 (27.7)	28 (57.1)	27.8±4.77	NA	NA	24 (38.7)	NA	NA	NA	14 (29.7)	7 (10.8)	NA	55 (90.2)	2 (3.3)	Stent thrombosis (n=65)
	64.8±12.7	138 (70.8)	146 (76.4	30 (17.5)	95 (64.6)	26.7±3.77	NA	NA	54 (29.5)	NA	NA	NA	11 (8.8)	7 (3.9)	NA	170 (93.4)	5 (2.7)	No stent thrombosis (n=195)
Auffret et al. (2016) [19]	68.0 (58.0-78.0)	234 (31.8)	360 (48.8	325 (44.1)	222 (30.1)	NA	NA	NA	88 (11.9)	9 (1.2)	NA	586 (88.5)	636 (96.1)	414 (62.5)	NA	NA	133 (20.3)	Stent thrombosis (n=737)
	68.0 (58.0-78.0)	252 (34.2)	355 (48.2)	329 (44.6)	220 (29.9	NA	NA	NA	90 (12.2)	7 (0.9	NA	667 (92.1)	697 (96.3)	427 (59.0)	NA	NA	159 (24.1)	No stent thrombosis (n=737)
Zwart et al. (2017) [20]	61.0 ±11.8	108/437 (24.7)	NA	NA	NA	NA	NA	NA	NA	500/866 (57.7)	NA	NA	NA	NA	NA	270 (61.8)	152 (34.8)	Stent thrombosis (n=437)
	62.3 ±11.7	235/862 (27.3)	NA	NA	NA	NA	NA	NA	NA	252/437 (57.7	NA	NA	NA	NA	NA	614 (70.9)	238 (27.5)	No stent thrombosis (n=866)
Rozemeijer et al. (2019) [21]	64.9±11.5	43 (78.2)	29 (52.7)	27 (49.1)	23 (41.8)	27.3±4.4	NA	10 (18.2)	16 (29.1)	6 (10.9)	29 (52.7)	NA	NA	NA	NA	26 (47.2)	29 (52.7)	Stent thrombosis (n=55)
	65.6±14.7	167 (75.9)	111 (51.8)	91 (41.3)	77 (35.0)	26.7±4.5	NA	44 (20.0)	40 (18.2)	21 (9.5)	24 (43.6)	NA	NA	NA	NA	88 (40.0)	132 (60.0)	No stent thrombosis (n=220)
Le Feuvre et al. (2008) [22]	60±14	49 (92)	28 (54)	NA	27 (52)	NA	6 (11)	NA	20 (38)	34 (65)/ 4 (8)	28 (54)	NA	NA	NA	NA	36/52	16 (31)	Stent thrombosis (n=52)
	63±11	2832 (80)	1799 (51)	NA	1353 (38)	NA	560 (16)	NA	1105 (31)	772 (22)/ 276 (8)	928 (26)	NA	NA	NA	NA	NA	1245 (35)	No stent thrombosis (n=3527)
Mishkel et al. (2008) [24]	56.6 ±16.3	27 (79.4)	NA	NA	19 (55.9)	13 (38.2)	NA	NA	10 (29.4)	9 (26.5)	17 (50.0)	NA	NA	NA	NA	NA	NA	Stent thrombosis (n=34)
	66.7 ±12.2	3302 (63.2)	NA	NA	1235 (23.6)	2671 (51.1)	NA	NA	1509 (28.9)	1220 (23.3)	1481 (28.3)	NA	NA	NA	NA	NA	NA	No stent thrombosis (n=5225)
Balghith et al. (2013) [25]	59	19 (100)	8 (42)	19 (52)	7 (37)	NA	NA	NA	9 (47)	NA	NA	NA	NA	NA	NA	4 (21)	15 (79)	Stent thrombosis (n=19)
Clemmensen et al. (2015) [26]	63 (54, 72)	5 (35.7)	2 (14.3)	3 (21.4)	3 (21.4)	NA	NA	NA	1 (7.1)	0 (0.0)	0 (0.0	NA	14 (100.0)	5/13 (38.5)	NA	NA	NA	Stent thrombosis (n=14)
	61 (62, 71	518 (23.7	961 (44.0)	812 (37.2)	922 (42.3)	NA	NA	NA	295 (13.5)	193 (8.8)	205 (9.4)	NA	2,181/2,183 (99.9	1,064/2,091 (50.9)	NA	NA	NA	No stent thrombosis (n=2184)
Driesman et al. (2015) [27]	57.4	8 (88.9)	3 (33.3)	3 (33.3)	3 (33.3)	NA	NA	NA	2 (22.2)	2 (22.2)	NA	NA	1 (11.1)	1 (11.1)	NA	5 (55.6)	3 (33.3)	Stent thrombosis (n=9)
	62.4	186 (70.7)	153 (58.2)	136 (51.9)	141 (56.6)	NA	NA	NA	44 (16.7)	57 (21.7)	NA	NA	88 (34.0)	16 (6.2)	NA	102 (38.8)	148 (56.3)	No stent thrombosis (n=263)
Brener et al. (2013) [28]	61.4 (52.4-69.5)	74.5 (35/47)	57.4 (27/47)	47.8% (22/46)	NA	NA	NA	86 (64-106)	17.0 (8/47)	14.9 (7/47)	17.0 (8/47)	NA	NA	NA	NA	34 (16/47)	66.0 (31/47)	Stent thrombosis (n=47)
	60.6 (52.7-70.0)	73.5 (4812/6544)	61.1% (3990/6529)	52.5% (3416/6501)	NA	NA	NA	90 (69-116)	24.2 (1581/6528)	22.7 (1463/6452)	29.3 (1913/6525)	NA	NA	NA	NA	19.1 (1248/6544)	80.9 (5296/6544)	No stent thrombosis (n=6544)
Lemesle et al. (2008) [23]	65.5 ±2.3	26 (66.7)	23 (59)	24 (61.5)	20 (51.3)	25.4 6 ±0.6	7 (17.9)	NA	11 (28.2)	NA	NA	NA	NA	NA	NA	NA	9 (23.1)	Stent thrombosis (n=39)
	67.1 6 ±2.8	18 (72)	15 (60)	16 (64)	13 (52)	24.5 6 ±0.8	4 (16)	NA	6 (24)	NA	NA	NA	NA	NA	NA	NA	6 (24)	No stent thrombosis (n=25)

Primary analysis

Abnormal Left Ventricular Ejection Fraction

**Figure 2:**
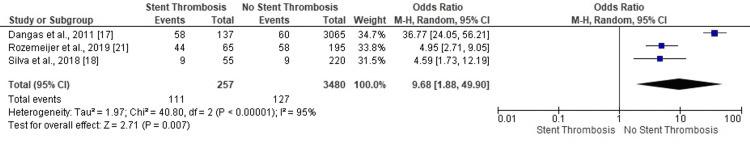
Forest plot (abnormal left ventricular ejection fraction) Image is created by the authors of this study.

Three studies revealed significant odds for no-ST in PCI patients with abnormal LVEF (OR: 4.59, 95% CI: 1.73-12.19; OR: 4.95, 95% CI: 2.71-9.05; OR: 36.77, 95% CI: 24.05-56.21) (Figure 2). The highly heterogeneous and statistically significant results favored no ST in PCI patients with abnormal LVEF (OR: 9.68, 95% CI: 1.88-49.90, p=0.007) (I^2^=95%, p<0.00001). 

Acute Coronary Syndrome Status

**Figure 3:**
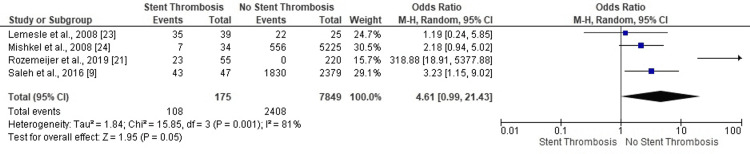
Forest plot (acute coronary syndrome status) Image is created by the authors of this study.

Four studies confirmed no attribution of acute coronary syndrome (ACS) for ST in PCI patients (OR: 1.19, 95% CI: 0.24-5.85; OR: 2.18, 95% CI: 0.94-5.02; OR: 3.23, 95% CI: 1.15-9.02; OR: 318.88, 95% CI: 18.91-5377.88) (Figure 3). The overall findings negated ST events in PCI patients with ACS status (OR: 4.61, 95% CI: 0.99-21.43, p=0.001). The results also proved highly heterogenous with a risk of publication bias based on the asymmetry of the corresponding funnel plot (I^2^=81%, p=0.001) 

*Antiplatelet Prescription* 

**Figure 4:**
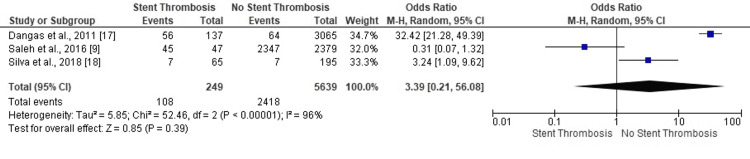
Forest plot (antiplatelet prescription) Image is created by the authors of this study.

Only one study confirmed the impact of antiplatelet therapy on ST in PCI scenarios (OR: 0.31, 95% CI: 0.07-1.32) (Figure 4). Two studies indicated no-ST in PCI patients with antiplatelet prescription (OR: 32.42, 95% CI: 21.28-49.39). The overall (statistically insignificant and highly heterogeneous) results confirmed no-ST in PCI patients who received antiplatelet therapy (OR: 3.39, 95% CI: 0.21-56.08, p=0.39) (I^2^=96%, p<0.00001). 

Aspirin Prescription

**Figure 5:**
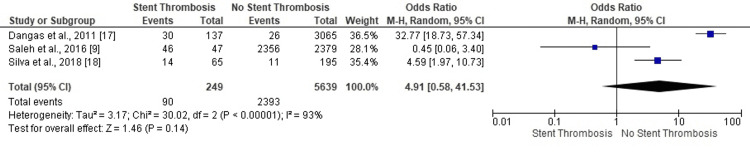
Forest plot (aspirin prescription) Image is created by the authors of this study.

One study reported ST in PCI patients with aspirin prescription (OR: 0.45, 95% CI: 0.06-3.40). Two studies confirmed the absence of ST in PCI patients who received aspirin therapy (OR: 32.77, 95% CI: 18.73-57.34; OR: 4.59, 95% CI: 1.97-10.73) (Figure 5). The highly heterogeneous results proved inconclusive for the predisposition of PCI patients (on aspirin prescription) to ST events (OR: 4.91, 95% CI: 0.58-41.53, p=0.14) (I^2^=93%, p<0.00001). 

*Diabetes Mellitus* 

**Figure 6:**
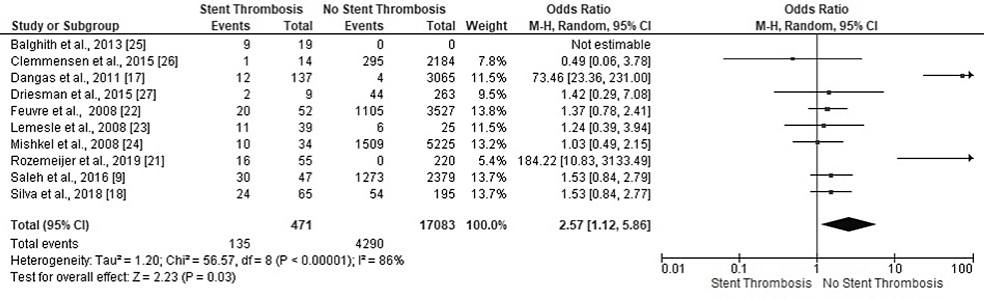
Forest plot (diabetes mellitus) Image is created by the authors of this study.

Seven studies affirmed no-ST in PCI patients with diabetes mellitus (DM) (Figure 6). Only one study revealed a statistically significant result confirming the development of ST in PCI patients with DM (OR: 0.49, 95% CI: 0.06-3.78). Six studies revealed lesser odds value for developing ST in PCI patients with DM. The highly heterogeneous findings conclusively negated the incidence of ST in DM patients with PCI (OR: 2.57, 95% CI: 1.12-5.86, p=0.03) (I^2^=86%, p<0.00001).

*Drug-Eluting Stent Status* 

**Figure 7:**
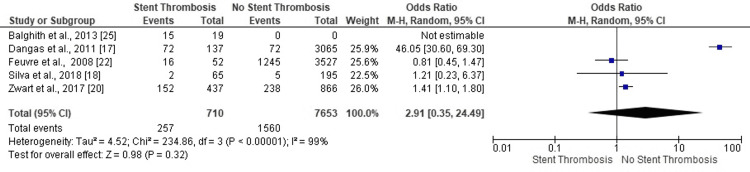
Forest plot (drug-eluting stent status) Image is created by the authors of this study.

Three studies confirmed no ST in PCI patients treated with drug-eluting stents (DES) (Figure 7). One study revealed odds for ST thrombosis in PCI scenarios based on DES (OR: 0.81, 95% CI: 0.45-1.47). The highly heterogeneous and statistically insignificant results proved inconclusive concerning no impact of drug stent status on ST events in PCI patients (OR, 2.91, 95% CI: 0.35-24.49, p=0.32) (I^2^=99%, p<0.00001). 

Dyslipidemia

**Figure 8:**
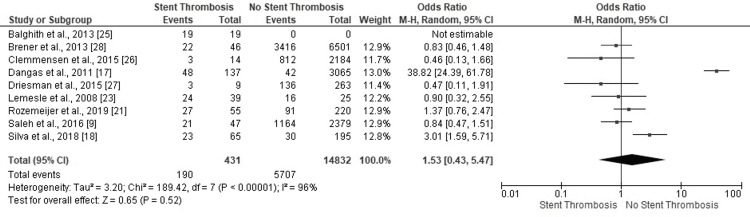
Forest plot (dyslipidemia) Image is created by the authors of this study.

Five studies revealed statistically insignificant findings confirming ST events in PCI patients with dyslipidemia (Figure 8). The highly heterogeneous and statistically insignificant results substantiated the incidence of ST among dyslipidemia patients with PCI (OR: 1.53, 95% CI: 0.43-5.47, p=0.52) (I^2^=96%, p<0.00001). The symmetrical funnel plot ruled out the scope of biased outcomes.

Elevated Cardiac Biomarkers

**Figure 9:**
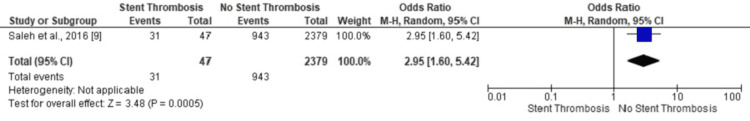
Forest plot (elevated cardiac biomarkers) Image is created by the authors of this study.

Our meta-analysis guided by a single study confirmed no-ST in PCI patients with elevated cardiac biomarkers (OR: 2.95, 95% CI: 1.60-5.42, p=0.0005) (Figure 9). However, the lack of data from other studies restricted the generalizability of this result. 

*Heart Failure* 

**Figure 10:**
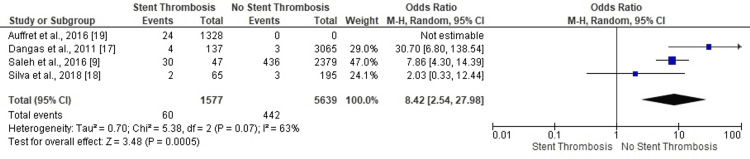
Forest plot (heart failure) Image is created by the authors of this study.

Three studies confirmed no ST in PCI patients with a clinical history of heart failure (OR: 8.42, 95% CI: 2.54-27.98, p=0.07) (Figure 10). The moderately heterogeneous results (I^2^=63%, p=0.07) were devoid of publication bias based on the symmetry of the corresponding funnel plot (Figure 10). 

History of MI or CAD

**Figure 11:**
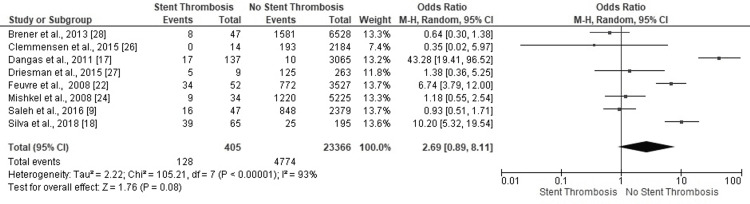
Forest plot (history of MI or CAD) Image is created by the authors of this study. MI: myocardial infarction; CAD: coronary artery disease

Three studies revealed ST in PCI patients with a clinical history of myocardial infarction (MI) or coronary artery disease (CAD) (Figure 11). Three studies reported substantial odds for no ST in similar scenarios. The highly heterogeneous findings conclusively favored no-ST events among PCI patients with MI or CAD (OR: 2.69, 95% CI: 0.89-8.11) (I^2^=93%, p<0.00001). 

*Hypertension* 

**Figure 12:**
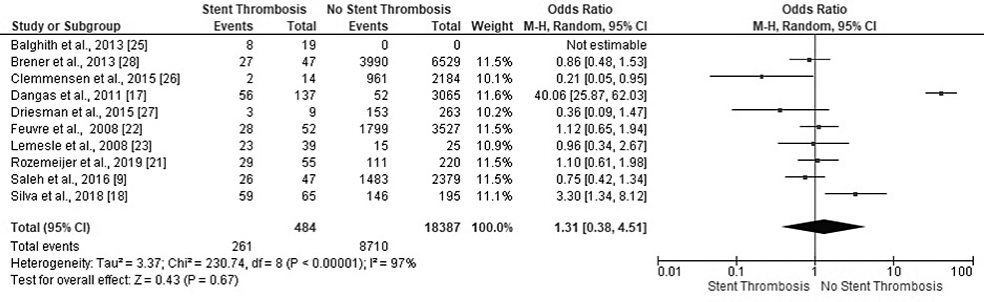
Forest plot (hypertension) Image is created by the authors of this study.

Six studies confirmed ST in PCI patients with hypertension. Two studies revealed greater odds for no ST in hypertensive PCI patients. The highly heterogeneous results concerning ST events in PCI patients with hypertension proved statistically insignificant and inconclusive (OR: 1.31, 95% CI: 0.38-4.51, p=0.67) (I^2^=97%, p<0.00001) (Figure 12). 

*Major Bleeding* 

**Figure 13:**
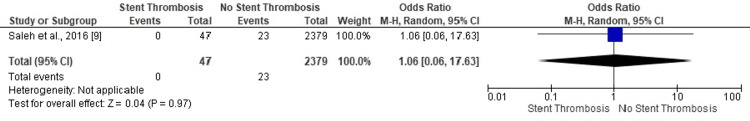
Forest plot (major bleeding) Image is created by the authors of this study.

One study produced a statistically insignificant effect size (Z=0.04) concerning the impact of major bleeding events on ST episodes in PCI patients (OR: 1.06, 95% CI: 0.06-17.63, p=0.97) (Figure 13). 

Mean Body Mass Index

**Figure 14:**
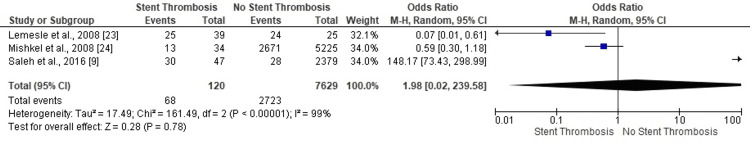
Forest plot (mean body mass index) Image is created by the authors of this study.

Two studies indicated ST in PCI patients with mean body mass index (BMI) (Figure 14). One study revealed substantial odds for no ST in a similar scenario. The overall results proved statistically insignificant and highly heterogenous for the impact of mean BMI on ST events in PCI patients (OR: 1.98, 95% CI: 0.02-239.58, p=0.78) (I^2^=99%, p=0.78). 

Percutaneous Coronary Intervention for Multivessel Coronary Artery Disease

**Figure 15:**
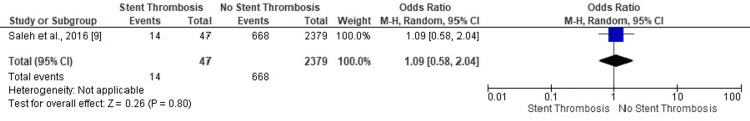
Forest plot (percutaneous coronary intervention for multivessel coronary artery disease) Image is created by the authors of this study.

One study produced a statistically insignificant effect size (Z=0.26) concerning the incidence of ST among patients with PCI for multivessel CAD (OR: 1.09, 95% CI: 0.58-2.04, p=0.80) (Figure 15). This finding remained questionable due to the lack of relevant data from other included studies. 

Prior History of Percutaneous Coronary Intervention

**Figure 16:**
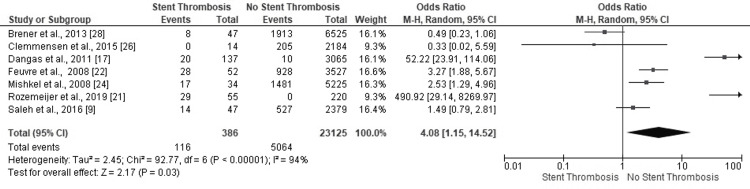
Forest plot (prior history of percutaneous coronary intervention) Image is created by the authors of this study.

Two studies revealed statistically significant odds for the occurrence of ST in PCI patients with a prior history of PCI (OR: 0.49, 95% CI: 0.23-1.06; OR: 0.33, 95% CI: 0.02-5.59, p=0.03) (Figure 16). However, the overall findings conclusively negated the events of ST in patients who underwent PCI in the past (OR: 4.08, 95% CI: 1.15, 14.52; p=0.03). The high heterogeneity of results was supported by the statistically significant I^2^ finding (94%, p<0.00001).

Renal Insufficiency

**Figure 17:**
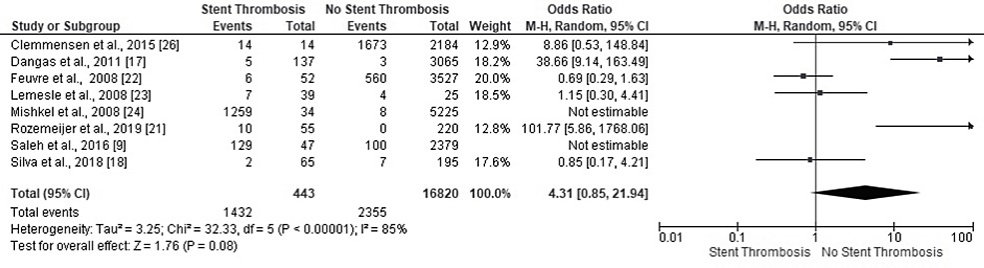
Forest plot (renal insufficiency) Image is created by the authors of this study.

Six studies affirmed no-ST in PCI patients with renal insufficiency. The overall (highly heterogenous) findings conclusively negated the correlation of ST events with renal insufficiency status of patients with PCI (OR: 4.31, 95% CI: 0.85-21.94, p=0.08) (I^2^=85%) (Figure 17). 

Smoking History

**Figure 18:**
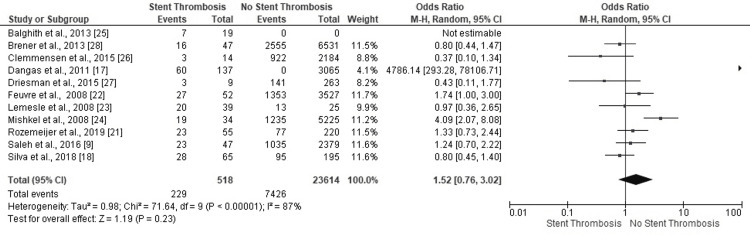
Forest plot (smoking history) Image is created by the authors of this study.

Four studies confirmed ST in PCI patients with a history of smoking (Figure 18). Six studies negated the development of ST in a similar scenario. The highly heterogeneous results, however, lacked statistical significance (OR: 1.52, 95% CI: 0.76-3.02, p=0.23) (I^2^=87%, p<0.00001). 

ST-Segment Elevation Myocardial Infarction

**Figure 19:**
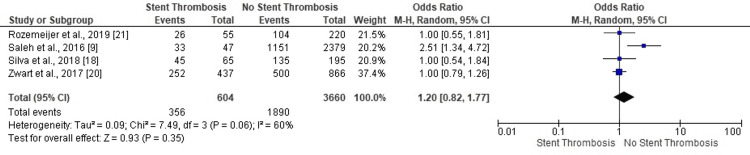
Forest plot (ST-segment elevation myocardial infarction) Image is created by the authors of this study.

One study confirmed no ST in PCI patients with ST-segment elevation myocardial infarction (STEMI) (Figure 19). The moderately heterogeneous results concerning ST in STEMI patients proved inconclusive and statistically insignificant (OR: 1.20, 95% CI: 0.82-1.77, p=0.35) (I^2^=60%, p=0.06). 

*Subgroup Analysis* 

Anterior Q waves: Two studies produced statistically insignificant results concerning the clinical correlation between anterior Q waves and ST in patients with PCI (OR: 0.97, 95% CI: 0.79, 1.20; p=0.78). The heterogeneity of the findings also lacked statistical significance (I^2^=0%, p=0.72). The symmetry of the funnel plot excluded the risk of publication bias. 

Atrial fibrillation: The finding from one study confirmed atrial fibrillation as the significant predictor of ST in PCI patients (OR: 0.55, 95% CI: 0.39, 0.77; p=0.0006). Another study revealed a contrary result confirming no-ST in PCI patients with atrial fibrillation (OR: 1.41, 95% CI: 0.40, 0.78; p=0.0006). The overall findings proved statistically significant and non-heterogenous (OR: 0.55, 95% CI: 0.40, 0.78; p=0.0006) (I^2^=0%, p=0.53). The symmetry of the funnel plot negated the scope of publication bias in the reported results. 

Bivalirudin monotherapy: Two studies produced statistically insignificant results for the clinical correlation of bivalirudin monotherapy with ST/no-ST events (OR: 1.20, 95% CI: 0.75, 1.94; p=0.45). The overall results proved moderately heterogeneous (I^2^=74%, p=0.05), while the funnel plot symmetry ruled out publication bias in the reported findings. 

Dual antiplatelet therapy: The findings from five studies substantiated the effectiveness of dual antiplatelet therapy (DAPT) in minimizing ST episodes in patients with PCI (OR: 1.08, 95% CI: 0.51, 2.30). The overall highly heterogeneous findings (I^2^=86%, p<0.00001) proved statistically insignificant based on the p-value of 0.83. The symmetrical funnel plot nullified publication bias in the reported results. 

In-stent diameter: The findings from one study correlated the in-stent diameter with the ST events in PCI patients (OR: 0.61, 95% CI: 0.11, 3.30). The statistically insignificant results favored no ST episodes in PCI patients based on their in-stent diameters (OR: 3.37, 95% CI: 0.28, 41.18; p=0.34). The findings proved highly heterogenous (I^2^=89%, p=0.0001), while the symmetry of the funnel plot negated the risk of biased outcomes. 

Lesion length/diameter: The findings from a single study correlated lesion length/diameter with ST events in PCI patients. Another study contrarily negated this relationship; however, the overall results proved statistically insignificant (OR: 0.36, 95% CI: 0.00, 38.51; p=0.67). The findings were highly heterogeneous (I^2^=98%, p<0.00001) with a high risk of publication bias. 

Lesion location (ostial or bifurcation lesion): The statistically insignificant findings from three studies correlated no ST with ostial/bifurcation lesion in PCI patients (OR: 1.22, 95% CI: 0.47, 3.19; p=0.69). The highly heterogeneous results (I^2^=91%, p<0.00001) excluded publication bias based on the symmetry of the funnel plot. 

Lesion target vessel (left anterior descending coronary artery): The non-heterogenous findings (I^2^=0%, p=0.96) from four studies were statistically insignificant and inconclusive for the incidence of ST/no-ST in PCI patients with left anterior descending coronary artery lesion (OR: 0.88, 95% CI: 0.69, 1.12). The funnel plot symmetry negated publication bias in the reported results. 

Lesion target vessel (left circumflex coronary artery; forest plot): Two studies revealed non-heterogenous (I^2^=0%, p=0.90) and statistically insignificant results for ST events in PCI patients with left circumflex coronary artery lesion (OR: 0.95, 95% CI: 0.69, 1.31). The findings were devoid of publication bias based on the funnel plot symmetry. 

Lesion target vessel (right coronary artery; forest plot): One study provided statistically insignificant results for ST events in PCI patients with right coronary artery lesions. The overall results were non-heterogenous (I^2^=0%, p=0.87) and inconclusive for the risk stratification of ST/no-ST episodes via right coronary artery lesion in the setting of PCI (OR: 1.05, 95% CI: 0.82, 1.34; p=0.71). The symmetrical funnel plot negated publication bias in the reported outcomes. 

Number of stents: Five studies indicated greater odds for no-ST in PCI patients based on the number of implanted stents. Four studies contrarily favored ST risk stratification (determined by the stent numbers) in PCI patients. The overall moderately heterogeneous findings (I^2^=64%, p=0.003) proved inconclusive for ST/no-ST episodes in the context of stent numbers in PCI scenarios (OR: 1.23, 95% CI: 0.63, 2.40; p=0.54). The symmetry of the funnel plot ruled out publication bias in the reported outcomes. 

Stent diameter (pre-procedure diameter stenosis): The findings from four studies conclusively substantiated no ST events in PCI patients with pre-procedure diameter stenosis (OR: 16.22, 95% CI: 3.06, 85.97; p=0.001). The results confirmed stent diameter as a significant risk stratification parameter in PCI scenarios. The overall outcomes proved highly heterogenous (I^2^=86%, p<0.0001), while the symmetry of the funnel plot negated the risk of publication bias. 

Stent length: One study conclusively correlated the ST events with the stent lengths in PCI scenarios. Five studies alternatively confirmed no ST in PCI patients based on their stent lengths. Stent length accordingly proved to be a significant predictor of ST/no-ST events in PCI patients (OR: 5.61, 95% CI: 1.62, 19.40; p=0.006). The highly heterogeneous findings (I^2^=93%, p<0.00001) were devoid of publication bias based on the symmetrical funnel plot. 

Thrombolysis in myocardial infarction flow: Three studies revealed thrombolysis in myocardial infarction (TIMI) flow as a marker to predict ST episodes in PCI patients. Four studies contrarily indicated no ST events in patients with PCI based on their TIMI flow. The overall findings proved inconclusive (OR: 1.04, 95% CI: 0.64, 1.70; p=0.87), moderately heterogeneous (I^2^=79%, p<0.00001), and without publication bias. 

Discussion

The findings from our systematic review revealed ST as a frequently reported condition in PCI patients (Table 1). Our meta-analysis, however, indicated statistically insignificant results concerning ST in PCI patients based on their antiplatelet/aspirin prescriptions, diabetes mellitus, drug-eluting stent status, dyslipidemia, history of MI/CAD, hypertension, mean BMI, renal insufficiency, and smoking history. Alternatively, most of the studies indicating no ST in PCI scenarios in the context of baseline characteristics also lacked statistical significance. Limited evidence revealed a statistically significant relationship between ST in PCI patients and their prior history of PCI/diabetes mellitus. The findings did not completely negate the ST attribution of these well-established characteristics; however, the overall outcomes did not statistically signify ST/no-ST causation of baseline characteristics in PCI scenarios [17-28]. The subgroup analysis indicated atrial fibrillation, stent diameter, and stent length as the significant predictors of ST/no-ST in PCI patients. The stent length and atrial fibrillation specifically predicted ST episodes, while stent diameter correlated with no-ST events in patients with PCI. 

Medical literature emphasizes the impact of intraprocedural ST on out-of-lab ST in PCI patients [28]. This finding reveals a high risk for ST in patients (with a previous history of ST) during PCI. Our findings align with these results since two of the included studies confirmed the development of ST in PCI patients with a previous history of PCI. The results, however, differ from the outcomes of a previously published study that affirmed the risk of very late ST in PCI patients treated with drug-eluting stents [29]. This difference is probably attributed to the lack of categorization of ST cases into early and late scenarios in our study. Prospective studies should further delineate the impact of drug-eluting stents on ST events in PCI patients. ST in many scenarios develops under the combined impact of postprocedural/procedural factors, lesion-related complications, and patient-related issues. The ST incidence rates of 1-2.2% across PCI patients with bare-metal/drug-eluting stents necessitate their intracoronary imaging to underline the predominant causes [30]. Furthermore, our findings selectively support current evidence concerning the impact of diabetes mellitus on the ST predisposition of PCI patients [12]. 

The frequently reported causative factors of ST include small vessel diffuse lesions, chronic occlusive lesions, bifurcation lesions, opening lesions, vascular graft lesions, restenosis lesions, intramural hematoma, vascular wall dissection, excessively long/overlapping multiple stents, incomplete stent expansion, and inadequate stent diameter [31]. The medication-related causes of ST include the premature discontinuation of clopidogrel sulfate, aspirin, or other antiplatelet medicines. Our findings, however, do not stratify the risk of ST in PCI based on the premature discontinuation of antiplatelet therapy. Medical literature recommends the assessment of very late ST risk scores in ACS cases with PCI [32]. Our findings contrarily negate ST risk stratification in PCI patients with ACS. The technical factors for ST include under-sized stents and bivalirudin therapy [33]. The impaired platelet responses of diabetic ACS patients with PCI also elevate their predisposition to ST [34]. These factors require a comprehensive assessment to further explore the ST attribution of baseline characteristics of PCI patients. 

Late stent failure often emanates due to atherogenesis (inside the neointima of the implanted coronary stent) and neo-atherosclerosis [35]. The limited half-life of antithrombotic medicines further leads to the reinduction of platelet aggregation/thrombosis within the coronary stent [36]. Periprocedural myocardial infarction in some scenarios also triggers ST after PCI [37]. Clinical studies continue exploring the mechanical factors that add to the hypo-responsiveness of PCI patients to antiplatelet therapy [38]. Future studies should further explore iatrogenic factors and their attribution to adenosine diphosphate (ADP) induced platelet aggregation in patients with PCI. ACS patients often require repeated PCIs to overcome their clinical complications attributed to distal-stent edge dissection and uncovered arteries. The initial coronary stenting, however, potentially elevates vagal baroreflex sensitivity of cardiac patients, eventually increasing their risk of hemodynamic complications, ST predisposition, and cardiac death [39]. In-stent restenosis in PCI scenarios develops due to myointimal trauma, neointimal hyperplasia, vascular remodeling, and elastic recoiling. Other possible factors adding to the risk of in-stent restenosis and subsequent ST include osteal lesions, vessel caliber, and genetic predisposition [40]. 

Limitations 

Firstly, the included studies provided moderate evidence concerning the correlation of ST events with baseline characteristics of PCI patients. Secondly, our systematic review did not investigate the pathophysiological mechanisms contributing to the onset of ST in PCI patients. Thirdly, the limited number of randomized controlled trials added to the possibility of selection bias and restricted the generalizability of our findings. Fourthly, our study did not examine the physiological mechanisms dominating the impact of baseline attributes on ST events in PCI scenarios. Fifthly, our study did not consider the role of environmental factors and comorbidities in triggering ST in patients with coronary stenting. Lastly, we did not delineate a time-based relationship of ST with baseline characteristics of PCI patients, which could have biased our overall results.

## Conclusions

Our findings confirmed a limited clinical significance of baseline characteristics of patients with PCI in determining their predisposition to ST. The results, however, supported the ST risk stratification potential of atrial fibrillation and procedural parameters, including stent length and stent diameter. However, the high heterogeneity in our outcomes questions their reliability in ST-related PCI scenarios. Our findings support the need to investigate relationships between baseline characteristics and procedural, medicinal, genetic, and other patient-related factors to unravel the pathological pathways of ST and its fatal complications in PCI patients. They also substantiate the need for developing adequate prediction models for ST episodes in patients undergoing PCI. The physiological pathways controlling the interaction of baseline characteristics of cardiac patients with their ST events are yet unknown and require comprehensive investigation through randomized controlled studies. Future studies should also evaluate the impact of restenosis, intramural hematoma, vascular graft lesions, vascular wall dissection, inadequate stent diameter, and premature discontinuation of antiplatelet medicines on ST episodes.
